# Angiogenin Expression during Early Human Placental Development; Association with Blood Vessel Formation

**DOI:** 10.1155/2014/781632

**Published:** 2014-07-01

**Authors:** Nadine Pavlov, Jean-Louis Frendo, Jean Guibourdenche, Séverine A. Degrelle, Danièle Evain-Brion, Josette Badet

**Affiliations:** ^1^INSERM, U1139, 75006 Paris, France; ^2^Université Paris Descartes, UMR S1139, Sorbonne Paris Cité, 75006 Paris, France; ^3^Université de Lorraine, 54000 Nancy, France; ^4^CNRS, UMR5547, Université de Toulouse 3, 31062 Toulouse, France; ^5^Clinical Endocrinology, Hôpital Cochin, AP-HP, Université Paris Descartes, 75006 Paris, France; ^6^PremUP Foundation, 75006 Paris, France; ^7^INSERM Unit 1139, Faculté des Sciences Pharmaceutiques et Biologiques, Université Paris Descartes, 4 Avenue de l'Observatoire, 75270 Paris, France

## Abstract

The placenta is a transient organ essential for fetal development. During human placental development, chorionic villi grow in coordination with a large capillary network resulting from both vasculogenesis and angiogenesis. Angiogenin is one of the most potent inducers of neovascularisation in experimental models *in vivo*. We and others have previously mapped angiogenin expression in the human term placenta. Here, we explored angiogenin involvement in early human placental development. We studied, angiogenin expression by *in situ* hybridisation and/or by RT-PCR in tissues and primary cultured trophoblastic cells and angiogenin cellular distribution by coimmunolabelling with cell markers: CD31 (PECAM-1), vascular endothelial cadherin (VE-cadherin), vascular endothelial growth factor receptor-2 (VEGF-R2), Tie-2, von Willebrand factor, CD34, erythropoeitin receptor (Epo-R), alpha-smooth muscle actin, CD45, cytokeratin 7, and Ki-67. Extravillous and villous cytotrophoblasts, isolated and differentiated *in vitro*, expressed and secreted angiogenin. Angiogenin was detected in villous trophoblastic layers, and structured and nascent fetal vessels. In decidua, it was expressed by glandular epithelial cells, vascular cells and macrophages. The observed pattern of angiogenin expression is compatible with a role in blood vessel formation and in cross-talk between trophoblasts and endothelial cells. In view of angiogenin properties, we suggest that angiogenin may participate in placental vasculogenesis and organogenesis.

## 1. Introduction

**Figure 1:**
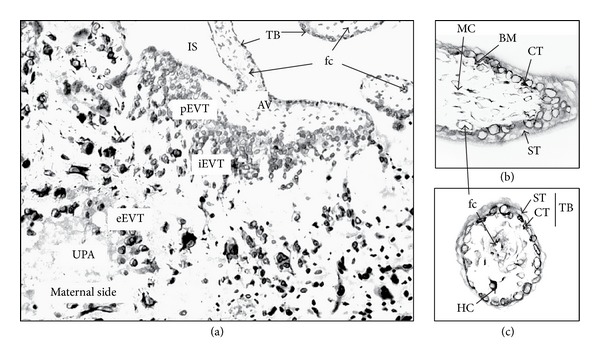
Schematic representation of an anchoring villus from a midgestational placental section, attached to the basal plate by a cell column (a). Schematic representation of a first-trimester mesenchymal villus: sagittal section (b) and cross-section (c). AV: anchoring villus; BM: basement membrane; CT: cytotrophoblast; eEVT, iEVT, and pEVT: endovascular-, interstitial-, and proliferating- extravillous trophoblast, respectively; fc: fetal capillary, HC: Hofbauer cell (macrophage); IS: intervillous space; MC: mesenchymal cell; ST, syncytiotrophoblast; TB: trophoblast layer; and UPA: uteroplacental artery.

The placenta is a transient organ essential for fetal development. It acts as an interface between the fetal and maternal blood circulation, ensuring (1) oxygen and nutrient transfer and waste removal, (2) immune protection and maternal tolerance of the semiallogeneic fetus, and (3) endocrine functions ([1, 2], for reviews). Chorionic villi are the essential structural and functional components of the human placenta (schematic representation in Figure 1). Their mesenchymal core is covered by a two-layered trophoblast epithelium that rests on a basement membrane in contact with the stromal core. This trophoblast layer is composed of villous cytotrophoblasts that proliferate and differentiate by fusion, forming a multinucleated syncytiotrophoblast that covers the entire surface of the villus, in direct contact with a maternal blood exudate during the first trimester of pregnancy and with maternal whole blood thereafter. The syncytiotrophoblast plays a major role in fetomaternal exchanges throughout pregnancy and in synthesis of steroid and peptide hormones required for fetal growth and development. A subset of chorionic villi anchors the placenta to the uterine wall. The base of these villi contains proliferating extravillous cytotrophoblasts aggregated in columns (Figure 1(a)). During the first and second trimesters, highly invasive extravillous cytotrophoblasts stop proliferating and invade the uterine interstitium, while maternal spiral arteries are remodelled into uteroplacental arteries. The maternal arterial endothelium, unlike the venous endothelium, is replaced by extravillous cytotrophoblasts which adopt an endothelial phenotype [3, 4]. This endo- and perivascular trophoblast invasion and arterial remodelling ensures optimal exchanges between the maternal and fetal circulation.

Angiogenin [5] is one of the most potent inducers of neovascularization in experimental models* in vivo* ([6, 7], for reviews). Its expression is developmentally regulated in rats and humans [8, 9]. We and others have previously shown that the secreted 14 kDa protein is expressed by placental cells in the human term placenta, including vascular, trophoblastic, and amnionic cells [10, 11]. Here, we further probed the role of angiogenin in placental development. Indeed, early placental villi start to develop as mesenchymal villi at week 5 of gestation, with fetal capillary segments being formed by vasculogenesis. These villi develop into immature intermediate villi from week 7 to 8. The capillary segments then fuse and elongate to form a simple capillary network. Starting at week 9, the preexisting capillary network expands by vasculogenesis and branching angiogenesis [12–16]. Decidual sections provide access to the maternal environment, site of intense morphological, physiological and immunological reorganizations. In this work, we examined the distribution and cellular sources of angiogenin in early human placental tissues and maternal decidua. Angiogenin transcripts were detected by* in situ* hybridisation in tissues and by RT-PCR in both tissues and primary cultures of villous trophoblasts. The protein was localized by immunofluorescence from 7.5- to 9-week placental cryosections, and its cellular distribution was established by dual immunolabeling with markers for trophoblastic, epithelial, mesenchymal, and endothelial cells; vascular smooth muscle cells; endothelial, hematopoietic, and erythroid precursors; leukocytes and mature monocytes; and proliferating cells. Angiogenin expression was also studied in primary cultures of first-trimester extravillous and villous trophoblasts. We interpreted our findings in view of recent knowledge of the biological activities of angiogenin.

## 2. Materials and Methods

### 2.1. Reagents

Aprotinin, DNase I, ovalbumin, and Triton X-100 were from Sigma Chemical Co. (St. Louis, MO). Tween 20 was from Merck (Darmstadt, Germany). Percoll was from Amersham Pharmacia (Uppsala, Sweden). Culture media, Hanks buffered saline solution (HBSS), and Hepes were from Gibco Laboratories (Grand Island, NY). Trypsin was from Difco Laboratories (Detroit, MI), and penicillin and streptomycin were from Invitrogen (Illkirch, France). Fetal bovine serum (FBS) was from Biological Industries (Kibbutz Beit Haemek, Israel) or PAA Laboratories GmbH (Les Mureaux, France). Sera were heat-inactivated before use. Paraformaldehyde (PFA) was from Electron Microscopy Sciences (Washington, PA). Antibodies used in the study are listed in Table 1. Normal serum from donkey or goat, human IgG, and IgG- and protease-free bovine serum albumin (BSA) were from Jackson ImmunoResearch (West Grove, PA). All chemicals were of analytical grade.

### 2.2. Tissue Collection

Human placental tissues from first-trimester pregnancies were collected after legal voluntary termination (7–14 weeks of gestation) at Broussais and Saint-Vincent de Paul hospitals (Paris, France). Second-trimester placentas were collected after medical termination for major fetal abnormalities [17]. Fetal karyotyping was normal. Term placentas (>37 weeks) were obtained after elective Caesarean section from healthy mothers with uncomplicated pregnancies delivered at Robert Debré, Saint-Vincent de Paul, and Tenon hospitals (AP-HP, Paris, France). The study was approved by our local ethics committee (CCPRB Paris Cochin no. 18-05) and the patients gave their informed consent. For immunofluorescence experiments and* in situ* hybridisation, pieces of placenta and decidua were embedded in Tissue-Tek O.C.T Compound (Sakura Finetek Europe, The Netherlands), frozen in isopentane, cooled with liquid nitrogen, and stored at −80°C until cryostat sectioning.

### 2.3. Villous and Extravillous Trophoblast Isolation and Primary Culture

Villous placental tissues were dissected free of membranes and vessels and then rinsed and minced in Ca^++^- and Mg^++^-free HBSS supplemented with 100 IU/mL penicillin and 100 *μ*g/mL streptomycin. Extravillous cytotrophoblasts were isolated by trypsin-DNase digestion as previously described [18]. Cells were plated on 7 *μ*g/cm^2^ Matrigel-coated dishes in DMEM/10% FBS/2 mM L-glutamine/25 mM Hepes/100 IU/mL penicillin/100 *μ*g/mL streptomycin (5-6 × 10^5^ cells/2 mL/8 cm^2^ culture dish) and maintained at 37°C in humidified 5% CO_2_/95% air (*n* = 8). Villous cytotrophoblasts were isolated with the method of Kliman et al. [19], essentially as previously described [20]. Cells were seeded in HAM F12/DMEM (vol./vol.) containing 10% FBS, 2 mM L-glutamine, 100 IU/mL penicillin, and 100 *μ*g/mL streptomycin (3.3 million cells/3 mL/20 cm^2^ culture dish) and maintained at 37°C in humidified 5% CO_2_/95% air (*n* = 11). The medium was changed daily for three days. The collected medium was centrifuged, frozen in liquid nitrogen, and stored at −20°C until use. In parallel experiments, cells were collected for RNA extraction. RT-PCR and ELISA studies (*n* = 3) were performed in triplicate.

### 2.4. Reverse Transcription-Polymerase Chain Reaction (RT-PCR)

Total RNA was extracted from frozen placental tissues with TRIzol reagent (Invitrogen SARL, Cergy Pontoise, France). Total RNA was isolated from cultured cells as described in the Atlas Pure Total RNA Labeling System user manual (Clontech Laboratories, Palo Alto, CA). The total RNA concentration was determined by absorbance at 260 nm and its integrity was checked by 1% agarose gel electrophoresis in the presence of ethidium bromide with UV visualisation.

First-strand complementary DNA was synthesised from 2 *μ*g of total RNA using 0.2 *μ*g oligo(dT)_15_ as primer (Promega Co., France) and SuperScript II RNase H Reverse Transcriptase (Invitrogen SARL) in a 20 *μ*L reaction volume as per the manufacturer's instructions. The synthesised cDNA was then subjected to PCR amplification as described in detail elsewhere [10]. Gene-specific primers (Table 2) were from Invitrogen SARL. Negative controls for RT-PCR lacked total RNA. Poly A+ RNA from human liver (Clontech laboratories, Inc.) was used as a positive control for angiogenin expression. The absence of contaminating DNA was confirmed by the obtention of a single RT-PCR product for *β*-actin, with the primers being located in different exons. In order to confirm that the PCR products are angiogenin amplicon, PCR products from angiogenin cDNA amplification were subjected to 2% agarose gel electrophoresis, extracted with JETsorb (GENOMED GmbH, Germany), cloned in the pCRII-TOPO plasmid vector (Invitrogen SARL), and sequenced using the ABI PRISM dRhodamine Terminator Cycle Sequencing Ready Reaction kit (Perkin Elmer Applied Biosystems).

### 2.5. *In Situ* Hybridisation on Placental and Decidual Cryosections

Angiogenin cDNA (BBG28, R&D systems, Abingdon, UK) was labelled by incorporation of digoxigenin-labelled dUTP by random priming with the DIG High Prime Labelling and Detection Kit I according to the manufacturer's recommendations (Roche Diagnostics, Meylan, France).

Seven-micrometer-thick frozen sections of placenta or decidua were mounted on Polysine slides (Menzel-Gläser, Germany) and then dried and fixed with 4% PFA for 40 min at 4°C. Remaining free reactive groups were blocked with 0.2% glycine (w/v). After four washes in PBS, the sections were dehydrated with graded ethanol solutions (30%, 2 × 50%, 70%, and 2 × 100%), rapidly air-dried, and stored at −80°C. After rehydration with graded ethanol solutions (100%, 70%, and 2 × 50%), the sections were digested with 1 *μ*g/mL Proteinase K (Sigma) in 20 mM Tris-HCl, 1 mM EDTA, and pH 7.6 for 15 min at 37°C. Postfixation was performed with 4% PFA for 20 min at 4°C, followed by blockade of remaining reactive groups with 0.2% glycine (w/v). The sections were prehybridised at 50°C for 4 hours in hybridisation buffer containing 5x standard saline citrate (SSC, Gibco), 0.1% N-lauroylsarcosine (Sigma), 20% blocking solution (Boehringer kit), 50% deionised formamide (Fluka Chemie, Buchs, Switzerland), and 0.02% SDS (Bioprobe, Montreuil, France). Hybridisation was performed overnight at 50°C in hybridisation buffer containing 0.25 ng/mL labelled angiogenin cDNA probe (BBG28). Sections were washed twice in 2x SSC for 5 min and twice in 1x SSC for 15 min, at 50°C. After saturating the sections with blocking buffer (Boehringer kit) for 45 min at room temperature, alkaline phosphatase-conjugated antidigoxigenin Fab (1 : 600) was added for 2 hours. Bound antibodies were revealed overnight by adding 5-bromo-4-chloro-3-indolyl phosphate (BCIP) and nitro-blue tetrazolium (NBT) in the presence of 2 mM levamisole to inhibit endogenous alkaline phosphatase activity. After counterstaining with Mayer's hematoxylin, the sections were mounted in Glycergel (Dako SA, Trappes, France). Negative controls were prepared either by pretreating the sections with 100 *μ*g/mL RNase A for 1 h at 37°C or by using a nonspecific digoxigenin-labelled DNA probe (pBR 328).

### 2.6. Immunoassay

The angiogenin immunoassay was finalised in our laboratory by using a rabbit polyclonal anti-angiogenin antibody as previously described [10] with some modifications. Microtiter plates (Immulon 4 HBX; Dynatech, Chantilly, France) were coated with 1.8 *μ*g/mL antiangiogenin IgG diluted in PBS (50 *μ*L) overnight at 4°C. After three washes with PBS, 0.5‰ Tween 20 (PBS/Tween), the plates were blocked with 0.1% gelatine, PBS/Tween for 1.5 h at room temperature. Solutions (250 *μ*L) containing either standard or samples buffered with 20 mM MOPS, pH 7.2, and diluted if necessary, were distributed and incubated overnight at 4°C in a moist chamber. The wells were then washed three times with PBS/Tween and blocked with 0.1% gelatine PBS/Tween for 1 h at room temperature. Then, 50 *μ*L of peroxidase-conjugated affinity-purified antiangiogenin IgG (5 *μ*g/mL in PBS/Tween containing 1% ovalbumin) was added for 2 h at 37°C. After three washes with PBS/Tween, bound antibody was revealed with O-phenylenediamine dihydrochloride (OPD) and H_2_O_2_; the reaction was stopped after 5 min by adding 4N H_2_SO_4_. Absorbance was determined at 490 nm in a microplate reader (Dynatech MR 5000). Normal human plasma was used as positive control. The detection limit was 7.8 pg/mL and the assay range was 7.8–500 pg/mL.

The human chorionic gonadotropin (hCG) concentration in conditioned media was determined by using an enzyme-linked fluorescence assay (Vidas System, Biomérieux, Marcy l'Etoile, France) with a detection limit of 2 mIU/mL.

### 2.7. Immunofluorescence Staining of Angiogenin in Cultured Trophoblasts

Cell cultures (*n* = 5) were washed twice with 60 mM Pipes/25 mM Hepes/10 mM EGTA/2 mM MgCl_2_, pH 6.9 (PHEM) at 37°C. Cells were fixed with 4% PFA in PHEM for 20 min at room temperature. After two washes with PHEM, remaining reactive groups were blocked by adding 50 mM NH_4_Cl in PBS for 10 min. Cells were washed twice in PBS and kept in PBS at 4°C until use. Immunofluorescence staining was performed on cells permeabilized with 0.5% Triton X-100 in PHEM for 2 min and then washed twice with PBS. Saturation was achieved with PBS, 10 mg/mL IgG-free BSA, 50 *μ*g/mL human IgG. The monoclonal anti-angiogenin antibody (Table 1) was diluted in the same buffer at 6 *μ*g/mL and incubated with the cells overnight at 4°C. After a sequence of five washes (two with PBS, one with PBS 0.1‰ Tween 20, and two with PBS) for 2 min, saturation was achieved with PBS, 5% goat serum, 5% donkey serum for 2 h at 4°C. The bound antibody was revealed by incubation for 2 h at room temperature with 3.5 *μ*g/mL FITC-conjugated goat anti-mouse IgM in PBS, 10 mg/mL BSA. After a sequence of five washes for 2 min and a quick wash in distilled water, the cells were mounted in Vectashield mounting medium with 4′,6-diamidino-2-phenylindole dihydrochloride (Dapi, Vector Laboratories, Burlingame, CA). In negative controls, the primary antibody was replaced by nonspecific mouse IgM.

### 2.8. Immunolocalization of Angiogenin in Human First-Trimester Placenta and Decidua Sections

Ten-micrometer-thick cryosections were mounted on Superfrost Plus slides and air-dried. The sections were then either fixed and permeabilised (chorionic villi) or left untreated (decidua) before immunodetection. After two washes in PBS, the sections were fixed either with 100% acetone for 10 min at −20°C (Figures 4(a) and 4(b)) or with 4% PFA in PBS for 20 min at 4°C. In the case of PFA fixation, remaining free reactive groups were blocked by adding 50 mM NH_4_Cl for 10 min. After two washes in PBS, chorionic villi cryosections were permeabilized by adding 0.5% Triton X-100 in PBS for 5 min. Following two washes with PBS, saturation was achieved with blocking buffer (PBS, 10% donkey or goat serum depending on the secondary antibody, 50 *μ*g/mL human IgG) for 30 min at room temperature. Angiogenin was immunodetected with either angiogenin-specific rabbit IgG or mouse IgM in blocking buffer overnight at 4°C. After 6 washes in PBS for 2 min, sections were incubated with blocking buffer for 30 min and bound antibody was revealed by incubation for 1.5 h in blocking buffer with a TRSC-conjugated donkey antibody against rabbit IgG, FITC-conjugated goat antibody against mouse IgM, or a TRITC-labelled goat antibody against rabbit IgG, at room temperature. Finally, the sections were washed six times with PBS and once with distilled water. Cryosections of chorionic villi were mounted in Vectashield mounting medium with Dapi, while decidual sections were mounted in Mowiol. Negative controls were generated by omitting the primary antibody or by using the same concentration of isotypic control immunoglobulin. In order to characterise the structures exhibiting angiogenin staining, antibodies raised against cell-type-specific markers (Table 1) were added to the incubation medium. Bound antibodies were revealed with FITC- or TRSC-conjugated secondary antibodies, the choice of fluorochrome depending on the antibody used for angiogenin detection (Table 1). The slides were examined with an Olympus phase-contrast microscope with a fluorescence attachment (BX-60). Images were captured with a Hamamatsu C4742-95 CCD camera and VisionStage VA software (Graftek, France). Immunolocalization studies (*n* = 22) were performed on cryosections from 5 placentas.

## 3. Results

### 3.1. Angiogenin Is Expressed throughout Human Placental Development

**Figure 2:**
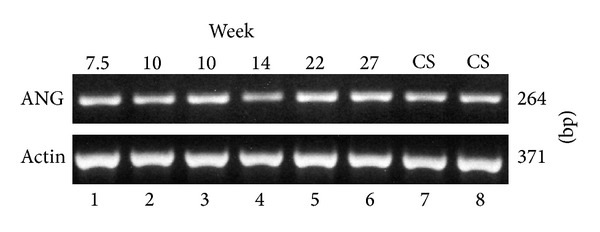
RT-PCR detection of angiogenin transcripts in human placenta. PCR products were obtained from first-trimester placenta RNA at weeks 7.5, 10, and 10: lanes 1, 2, and 3, respectively; from second-trimester placenta RNA at weeks 14, 22, and 27: lanes 4, 5, and 6, respectively; from term placenta RNA: lanes 7 and 8.

Angiogenin transcripts were detected by RT-PCR in placental tissues from 7.5 weeks gestation to term (Figure 2).

### 3.2. Angiogenin Is Expressed by Isolated Trophoblasts* In Vitro*

Extravillous cytotrophoblasts were cultured from first-trimester placentas. Angiogenin was detected with a sandwich ELISA in conditioned media from six of eight cultures. The angiogenin concentration after 48 h of culture ranged from 10 to 255 pg/mL.

**Figure 3:**
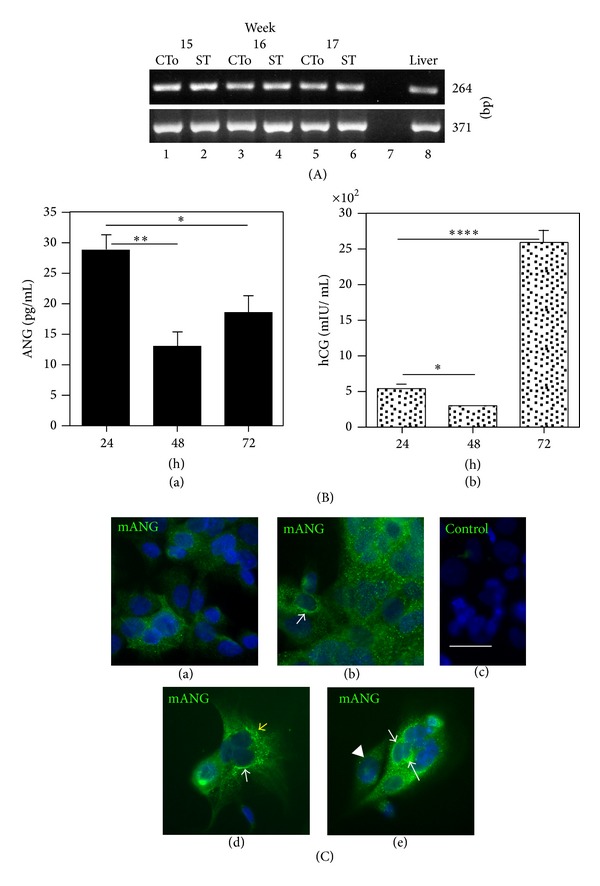
Angiogenin expression by trophoblastic cells. (A) Expression of angiogenin transcripts by trophoblasts isolated from second-trimester placenta. RT-PCR detection of a 264 bp fragment of the angiogenin transcript on 2% agarose gel stained with ethidium bromide. Lanes 1, 3, and 5: cytotrophoblasts from 15-, 16-, and 17-week placentas prior to culture (CT0) and 2, 4, and 6: RNA from 72 h cultured cytotrophoblasts, respectively, differentiated* in vitro* into a syncytiotrophoblast (ST); 7 is a negative control without RNA; 8 is a positive control using human liver RNA. *β*-actin PCR gave a product at 371 bp. (B) Angiogenin release by cultured villous cytotrophoblasts from 14-week placenta. Angiogenin (ANG) was released into the culture medium during* in vitro* differentiation of cytotrophoblasts into a syncytiotrophoblast (a). Human choriogonadotropin (hCG) was maximally expressed on day 3, indicating a functional syncytium (b). Results are means ± SD of triplicate determinations in a representative experiment (*P < 0.05; **P < 0.01; ****P < 0.0001). (C) Angiogenin immunodetection in cultured cytotrophoblasts from first-trimester chorionic villi* in vitro*. The cells were fixed with paraformaldehyde and permeabilized and then reacted with monoclonal antiangiogenin. The bound antibody was revealed with FITC-conjugated goat anti-mouse IgM (mANG, in green). Nuclei were counterstained with Dapi (in blue). Angiogenin staining increased with cell differentiation: villous cytotrophoblasts at day 1 (a) compared to the cells at day 2 (b); control with nonspecific mouse IgM was negative (c). Angiogenin labelling was heterogeneous: diffuse in single cells (e, arrowhead), dense and more pronounced around nuclei in aggregating cells (b, e, and white arrow), punctuated and associated with granules here in the syncytium (d, yellow arrow). Cells were from 13-week (a, b, and c), 12.5-week (d), and 8.5-week (e) placenta, respectively. Bar, 20 *μ*m.

Villous cytotrophoblasts were isolated and cultured from first- and second-trimester placentas. They did not proliferate but aggregated, fused, and formed a functional endocrine syncytium* in vitro*, secreting hCG as shown in Figure 3(B)(b) [20]. Angiogenin transcripts were detected by RT-PCR in freshly isolated trophoblasts, as well as in functional syncytiotrophoblasts produced* in vitro* (Figure 3(A)). Angiogenin was detected by ELISA in conditioned media throughout the differentiation process (Figure 3(B)(a)). Angiogenin immunostaining intensity increased with cell differentiation (Figure 3(C)). Labelling was cytoplasmic, with marked heterogeneity across single, aggregated, and fused cells. Angiogenin staining was either diffuse (more pronounced around nuclei) or located in granules. Staining specificity was shown by the negativity of controls (Figure 3(C)(c)).

### 3.3. Angiogenin Is Immunodetected* In Situ* in First-Trimester Chorionic Villi

**Figure 4:**
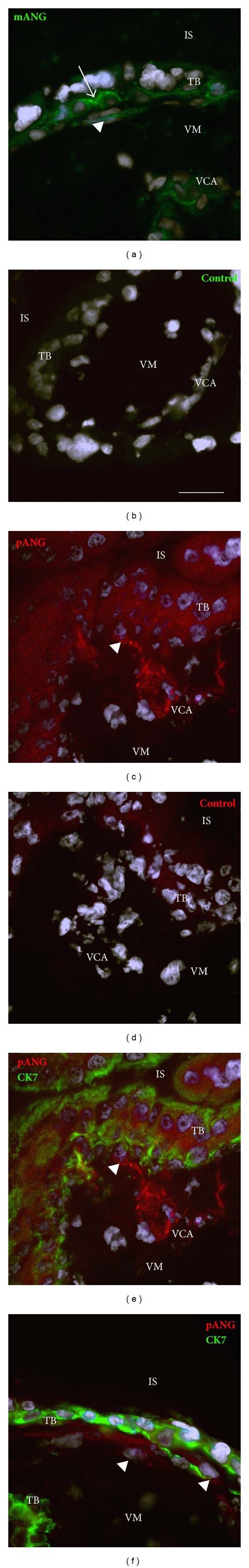
Angiogenin immunoreactivity in chorionic villi from human first-trimester placentas. Frozen sections of placentas at week 8 to 9 were reacted with either a monoclonal antibody against angiogenin (a, mANG in green) or a polyclonal antibody purified by affinity on immobilised angiogenin (c, e, f, and pANG in red). Bound antibodies were detected with fluorescent secondary antibodies. Angiogenin immunoreactivity (pANG, in red) was detected in the trophoblastic layer immunostained for cytokeratin 7 (CK7, in green) (e, f), in some cells in the close vicinity of the layer (arrowhead: a, in green; c, e, and f, in red) and in isolated cell masses (VCA), located deeper in the villous stroma (a, in green; c and e, in red). The signal was stronger in some cytotrophoblasts (arrow: a). No signal was observed on cryosections reacted with either nonspecific mouse IgM (control, b) or nonspecific rabbit IgG (control, d). Nuclei were counterstained with Dapi, in blue. Bar, 20 *μ*m. IS: intervillous space; TB: trophoblast layer; VCA: vascular cell aggregate; and VM: villous mesenchyme.

The location of angiogenin was studied by indirect immunolocalization on cryosections of human 8- to 9-week placentas, using two angiogenin-specific antibodies: a mouse monoclonal antibody (mANG) and a rabbit polyclonal antibody (pANG). Schematic villus sagittal sections and cross-sections are presented in Figures 1(b) and 1(c). A similar labelling pattern was obtained with the two antibodies: it covered the trophoblastic layer (identified by cytokeratin 7 (CK7) staining) and cells located within the villous stroma (Figure 4). Marked heterogeneity in the labelling intensity was observed from one villous to another and also within the villous itself (Figure 4(a)).

The trophoblastic layer was stained specifically for CK7 (Figures 4(e) and 4(f)) and was typically organised, with the syncytiotrophoblast lying on a continuous cytotrophoblastic layer (Figure 4(e)) in contact with the trophoblastic basement membrane (not visible here). In some villi, the trophoblastic layer was thinner and straightened (Figure 4(f)), as observed in term villi. In a few places, CK7-positive cells seemed to plunge from the trophoblastic layer into the core of the villi (Figures 4(e) and 4(f)). The villous trophoblastic layer was always immunolabelled for angiogenin, either uniformly (Figure 4(c)) or more strongly in some cytotrophoblasts (Figure 4(a)).

In the villous stroma, angiogenin was detected in some single cells close to the trophoblastic layer (Figures 4(a), 4(c), 4(e), and 4(f), arrowhead) and also in cell masses located deeper in the villous stroma (Figures 4(a) and 4(e)) (see Section 3.5). No signal was observed in negative controls (Figures 4(b) and 4(d)).

### 3.4. Angiogenin Is Expressed in the Decidua at Week 7.5 (Glandular Epithelium, Decidual Cells, Maternal Artery, and Macrophages)

**Figure 5:**
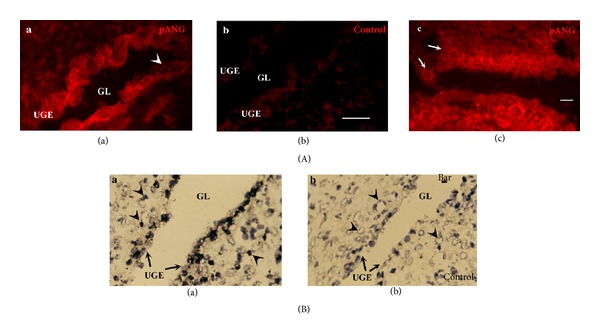
Angiogenin expression in parietal decidua: glandular epithelium and decidual cells. (A) Angiogenin immunoreactivity on frozen sections at week 7.5, using an angiogenin-specific polyclonal antibody; the glandular epithelium showed punctuate signal corresponding to secretory granules (a, arrowhead) and strongly delineated polyhedral decidual cells (c, arrows). No signal was observed on cryosections which reacted with nonspecific rabbit IgG (b). (B) Angiogenin transcripts were detected in the glandular epithelium (a, arrows) and in endothelial cells of small capillaries (a, arrowhead) on frozen sections hybridised with a digoxigenin-labelled angiogenin cDNA probe. Probe binding was detected with an alkaline phosphatase-coupled antibody to digoxigenin and visualised with a colorimetric substrate (NBT/BCIP) (a). No signal was observed in RNase pretreated frozen sections (b). Counterstaining with Mayer's haematoxylin. Bar, 20 *μ*m. GL, glandular lumen and UGE, uterine glandular epithelium.

**Figure 6:**
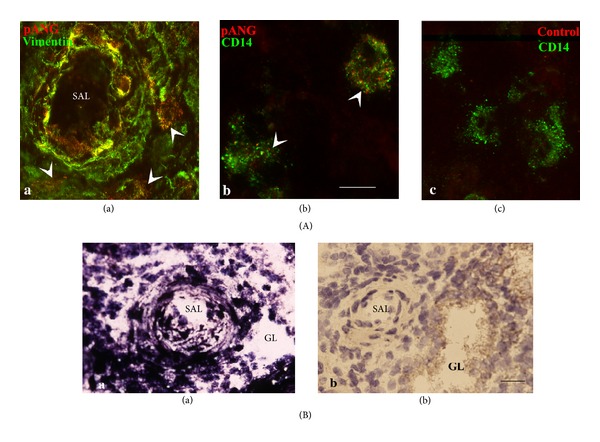
Angiogenin expression in decidua: maternal artery and macrophages. (A) Angiogenin immunoreactivity on frozen sections at week 7.5: (a) angiogenin specific polyclonal antibody (in red) highlighted vimentin-positive cells (in green): endothelial cells of the spiral artery (in yellow (red + green)) and sparse cells in the decidua (arrowhead). (b) Maternal macrophages (CD14 positive, in green) in a section of 11-week placenta were immunolabelled for angiogenin (in red), compared with the isotypic negative control (c). (B)* In situ* hybridisation on a frozen section of a 10-week placenta with a digoxigenin-labelled angiogenin cDNA probe revealing (in purple) angiogenin transcripts in spiral artery, glandular epithelium, and unidentified decidual cells (a). No signal was observed in RNase pretreated frozen sections (b). Counterstaining with Mayer's haematoxylin. Bar, 20 *μ*m. GL: glandular lumen and SAL: spiral artery lumen.

Tissue pieces were identified as parietal decidua based on the absence of invading cytotrophoblasts (CK7-positive cells) and remodelled spiral arteries. Uterine glands were present on the cryosections (Figure 5). Glandular epithelial cells, positive for cytokeratin (not shown), were immunolabelled for angiogenin. The intense, punctuate signal thus obtained likely corresponded to secretory vesicles (Figure 5A(a), arrowhead). No signal was observed in the control (Figure 5A(b)). Angiogenin was also detected on the outline of polyhedral decidual cells (Figure 5A(c), arrows). Angiogenin messengers were expressed by glandular epithelial cells and in endothelial cells of small maternal capillaries, as shown by* in situ* hybridisation (Figure 5B(a)). No signal was observed on negative controls either pretreated with RNase (Figure 5B(b)) or reacting with a nonspecific probe (not shown). The wall of spiral arteries was immunolabelled for angiogenin (Figure 6A(a)). Specific punctuate labelling was also observed in vimentin-positive cells (Figure 6A(a), arrowheads). The latter cells, in close contact with spiral arteries or sparser in the decidua, were shown to be macrophages, based on their CD14 reactivity (Figure 6A(b)). Thus, maternal macrophages were immunolabelled for angiogenin. Angiogenin transcripts were detected in spiral arteries by* in situ* hybridisation (Figure 6B(a)). Angiogenin transcripts were also strongly detected in unidentified small round cells. Negative controls demonstrated the specificity of the signal (Figure 6B(b)).

### 3.5. Angiogenin Expression Is Associated with First-Trimester Chorionic Villi and Blood Vessel Formation

**Figure 7:**
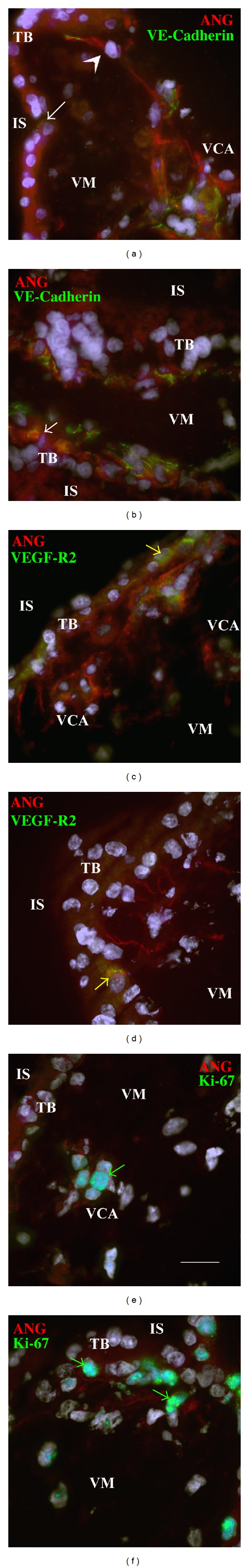
Characterisation by double labelling of chorionic villi undergoing intense morphological changes. Frozen cross-sections of human chorionic villi at week 8 were reacted with a mix of two primary antibodies. Angiogenin was detected with a specific polyclonal antibody (ANG, in red). The other antibody (in green) was directed against either the early endothelial markers: VE-cadherin (a, b) and VEGF-R2 (c, d), or the proliferation marker Ki-67 (e, f). Cells positive for the endothelial markers were stained for angiogenin. Note the angiogenin- and VE-cadherin-positive single cell (a, arrowhead) beneath the trophoblastic layer establishing a bridge between the layer and a vascular cell aggregate (VCA) stained for VE-cadherin; VEGF-R2-labelled VCA in close contact with the trophoblast layer (c); and angiogenin-labelled cytoplasmic processes (d). Proliferative cells labelled for Ki-67 and angiogenin were observed in aggregates (e, green arrow). Coordinated sites of proliferation (Ki-67+) composed of villous trophoblasts and facing nontrophoblastic cell were positive for angiogenin (f, green arrow). White and yellow arrows point to VE-cadherin and VEGF-R2 staining of trophoblast layer, respectively. Nuclei were counterstained with Dapi, in blue. Bar, 20 *μ*m. IS: intervillous space; TB: trophoblast layer; VCA: vascular cell aggregate; and VM: villous mesenchyme.

**Figure 8:**
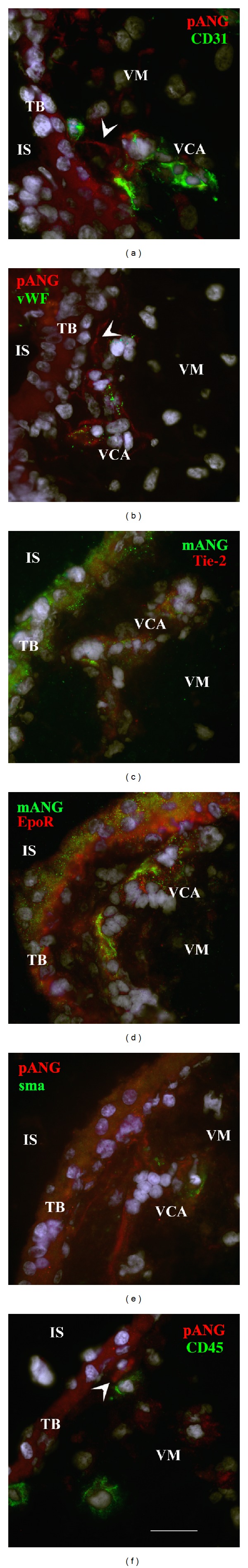
Immunolocalization of angiogenin in chorionic villi showing vascular cell aggregates. Frozen cross-sections of human chorionic villi at week 8 were reacted with a mix of two primary antibodies. The bound antibodies were detected with fluorescent secondary antibodies. The anti-angiogenin antibody was either polyclonal (a, b, e, f, and pANG in red) or monoclonal (c, d, and mANG in green). Angiogenin colabelling with CD31 (a, in green), vWF (b, in green), Tie2 (c, in red), Epo-R (d, in red), and *α*-smooth muscle actin (e, sma, in green), respectively, stained vascular cell aggregates. Arrowheads point to tiny cytoplasmic processes in close vicinity with the trophoblast layer connected with vascular cell aggregates (a, b). Angiogenin immunoreactivity was also detected in the trophoblastic layer, in cytotrophoblasts (also labelled for EpoR (d, in red)) and in single cells close to CD45-positive cells (f, in green, arrowhead). Nuclei were counterstained with Dapi, in blue. Bar, 20 *μ*m. IS: intervillous space and TB: trophoblast.

**Figure 9:**
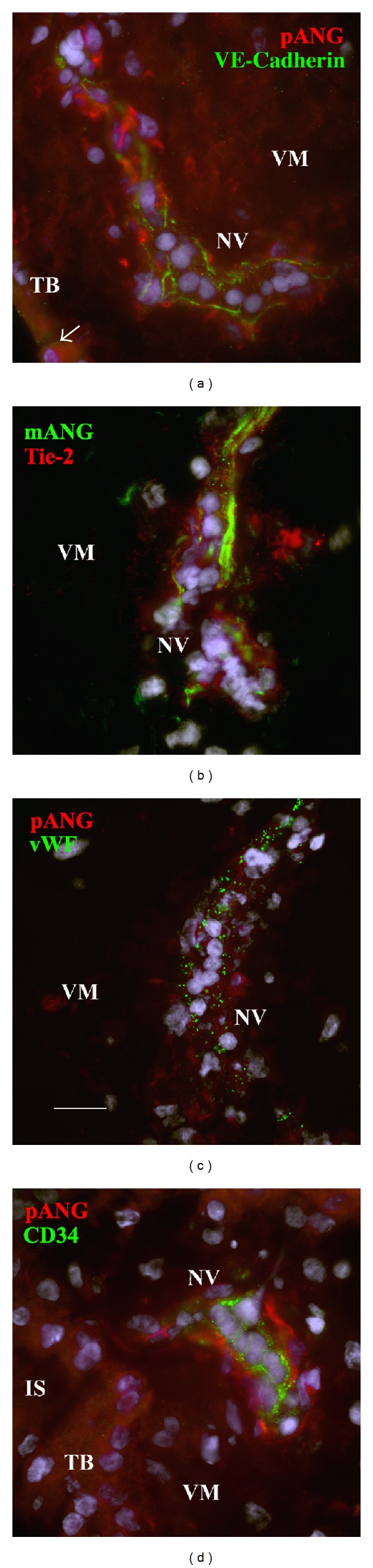
Angiogenin immunoreactivity in nascent vessels. Frozen sections prepared at week 8 were reacted with either a polyclonal antibody specific for angiogenin (a, c, d, and pANG in red) or a monoclonal (b, mANG in green). The other antibody, directed against VE-cadherin (a, in green), Tie2 (b, in red), vWF (c, in green), and CD34 (d, in green) colabelled nascent vessels. Nuclei were counterstained with Dapi, in blue. Bar, 20 *μ*m. IS: intervillous space; TB: trophoblast layer; VCA: vascular cell aggregate; and VM: villous mesenchyme.

The structure of the fetal vessels in the villus followed a gradient: the least organised vessels were close to the trophoblastic layer, while more organised ones were located deeper in the villus. In order to identify the cells that were immunopositive for angiogenin in developing chorionic villi, double immunolabelling with cell markers was performed on 8-week cryosections. Besides the trophoblastic layer (Figure 4), angiogenin staining was associated with three different structures: single cells, cell aggregates, and cell cords (Figures 7–9). Angiogenin labelling was associated with chorionic villi undergoing intense morphological changes (Figure 7). The labelled cells in close proximity of the trophoblastic layer expressed very early endothelial markers such as VE-cadherin (Figures 7(a) and 7(b)) and VEGF-R2 (Figures 7(c) and 7(d)).  Figure 7(a) shows a typical view of a single cell double stained for angiogenin and VE-cadherin establishing a bridge between cytotrophoblasts and cell aggregates (double stained), corresponding to a nascent fetal blood vessel. Proliferative cells labelled for Ki-67 antigen were observed in aggregates (Figure 7(e)). In less organised aggregates, Ki-67 was present both in some trophoblastic nuclei and in nuclei of underlying nontrophoblastic cells that were positive for angiogenin (Figure 7(f)). Tiny cytoplasmic processes, which are also labelled for angiogenin, were in intimate contact with the trophoblastic layer and connected with primitive established vessels in the core of the villi (Figures 8(a) and 8(b), arrowheads). Colabelling not only with CD31, an early endothelial marker, but also with* vWF*, a later endothelial marker, was shown in Figures 8(a) and 8(b), respectively. These observations suggest the existence of coordinated sites of proliferation composed of villous trophoblasts and facing nontrophoblastic cells. In cell aggregates, double staining for angiogenin and Tie-2, an endothelial marker, was observed (Figure 8(c)). As in term placenta, angiogenin and erythropoietin-receptor (EpoR) labelling were colocalized in the trophoblastic layer and in nascent or established fetal vessels (Figure 8(d)). However, EpoR labelling appeared stronger in cytotrophoblasts. In cell aggregates, colabelling with alpha-smooth muscle actin, a marker for vascular smooth muscle cells and pericytes, was detected (Figure 8(e)).

Angiogenin immunolabelling was observed in cords positive for the following early endothelial markers: VE-cadherin, CD34, and Tie-2 or for* vWF*, a later vascular marker (Figure 9). These nascent structures, corresponding to fetal vessels in formation and previously called angioblastic strands [23] or haemangioblastic cell cords [14], were located in a peripheral position in the villus. CD34-positive cells with rounded nuclei were present in these vessel segments (Figure 9(d)) and likely corresponded to haematopoietic precursors [24].

In the villous core, fetal macrophages (Hofbauer cells) were immunoreactive for CD45 (Figure 8(f)) but negative for CD14 (data not shown). The CD45-positive cells were angiogenin-negative but lay close to angiogenin-positive mesenchymal cells or in the close vicinity of the trophoblastic layer which was labelled strongly for angiogenin (Figure 8(f)).

In some villi, the trophoblastic layer was labelled for early endothelial cell markers such as VE-cadherin on its basal side (Figures 7(a) and 7(b) and Figure 9(a), arrows) and also for VEGF-R2 (Figures 7(c) and 7(d), arrows) but not for CD31 (Figure 8(a)), CD34 (Figure 9(d)), Tie-2 (Figure 8(c)), or* vWF* (Figure 8(b)). In areas labelled for both angiogenin and VE-cadherin, the trophoblastic layer was thickened by superimposed nuclei (Figure 7(b)). Unlike in term villi, the trophoblastic basement membrane of first-trimester villi was not highlighted by angiogenin immunolabelling.

Taken together, these results show that angiogenin protein is present in the villous trophoblastic layer, structured fetal vessels, and sites of nascent fetal vessels.

## 4. Discussion

Vessel formation during human placental development occurs by means of both vasculogenesis (from* in situ* differentiating endothelial cells) and angiogenesis (sprouting of capillaries from existing vessels) [14, 25]. Fetoplacental vasculogenesis proceeds by formation of haemangioblastic cords which progressively linked up from day 22 to the 26th week [14, 26, 27]. Current knowledge of the cellular and molecular mechanisms involved in human placental blood vessel formation relies mainly on morphological and ultrastructural observations [14, 23, 28] and immunohistochemistry [27, 29, 30], but further insights are being obtained by the study of angiogenic growth factors [10, 30–33] and molecular methods [2].

Angiogenin has been first isolated from supernatants of colon carcinoma cells on its property to induce angiogenesis in chicken chorioallantoic membrane [5]. Angiogenin (RNase 5) belongs to the secreted RNase family, which is vertebrate-specific and displays weak ribonucleolytic activity. An intact catalytic site and cell-binding domain are both required to induce neovascularization [34]. Angiogenin is also a permissive factor for angiogenesis induced by other angiogenic factors such as vascular endothelial growth factor (VEGF) and fibroblast growth factor (FGF-1 and FGF-2) [35], stimulating rRNA transcription and proliferation [36]. Among its other properties, angiogenin promotes cell survival, a property related to its ability to cleave tRNA and release tiRNAs (stress-induced small RNAs), which inhibit protein translation [37–40]. Angiogenin-induced tiRNAs promote stress granule assembly [37], an adaptive process that reprograms protein translation during stress [41, 42].

In this work, in agreement with previous observations of early placentas [30, 33, 43–45], we observed* in situ* immunostaining for vessel markers on single cells in not only close contact with the trophoblastic layer but also deeper in the villous stroma, on cell aggregates referred to as angioblasts by Florence Sabin in the chick [46]. Both sites were strongly labelled for angiogenin. The current consensus is that angioblastic cords arise from* in situ* differentiation of stem cells present in the embryonic-derived mesoderm that has invaded the chorionic villi [47]. These structures were found here to express endothelium-specific markers at 8 weeks of gestation, namely VE-cadherin, CD31 (PECAM-1, also present in leukocytes and platelets), VEGF-R2, Tie-2, vWF, CD34 (also present on haematopoietic stem cells), EpoR (also present on erythroid precursors), and angiogenin. Some of them also expressed the smooth muscle cell marker alpha-actin, suggesting the presence of pericytes.

Single cells in close contact with the trophoblastic layer were found to express VE-cadherin and PECAM-1 and also showed strong angiogenin expression. They were negative for the trophoblast marker CK7 and for the leukocyte differentiation antigens CD14 and CD45. Arthur Hertig suggested that these single cells might derive from trophoblast delamination and differentiation [23]. However, ultrastructural studies showing a continuous trophoblastic basal lamina throughout this early stage of development argue against this hypothesis [14, 28, 48]. It might be reconsidered [49] since epithelial-mesenchymal transitions are part of the developmental program [50] and the human cytotrophoblastic cell has been qualified as a “mononuclear chameleon” [51]. In certain areas, we observed trophoblastic expression of early endothelial markers such as VE-cadherin and VEGFR-2, as previously described [30, 33]. A switch in cadherin expression has been linked to cytotrophoblast differentiation [4]. However, this epithelial-/endothelial-like conversion has been only described for invasive cytotrophoblasts [52]. In any case, we observed that these cells in close contact with trophoblasts and the cellular extensions that bridge the cytotrophoblast layer to nascent vessels expressed endothelial markers. They were also strongly labelled for angiogenin as were the trophoblasts facing them. Together, these observations point to the existence of paracrine or juxtacrine interactions involving angiogenin. Paracrine interaction has been shown to mediate angiogenin neuroprotection. Skorupa et al. [53] have shown that angiogenin secreted by motoneurons is endocytosed by astroglia via syndecan 4. Interestingly, the paracrine RNA fragments in astrocytes differ from those previously described but their biological function is unknown. These observations from studies of the nervous system highlight the fundamental properties of angiogenin.

Angiogenin immunolabelling in early chorionic villi was associated with actively dividing endothelial and trophoblastic cells, possibly reflecting coordinated morphological development. In early placenta, vasculogenesis might be controlled by villous cytotrophoblast [30]. In this work, angiogenin was expressed by cytotrophoblasts. The fact that angiogenin binds to endothelial cells* via* high affinity binding sites [54] and stimulates proliferation [55, 56] and differentiation* in vitro *[57] suggests that cytotrophoblasts might release angiogenin as a paracrine signal to endothelial cells. Reciprocally endothelial cells might signal to trophoblastic cells in early villus development. It has been shown in developing organs such as liver, pancreas, or prostate that early endothelial cells and nascent vascular structures provide developmental signals to the growing organ throughout its development, even prior to blood vessel formation [58, 59]. In this work, angiogenin expression observed* in situ* corroborates its expression by endothelial cells* in vitro* [60]. However, evidence for angiogenin receptor on cytotrophoblasts is not yet documented. Taken together, these observations suggest cooperation between villous trophoblasts and nascent fetal blood vessels.

Unlike term placenta, the villous tree in first-trimester placenta (up to 10–12 weeks of gestation) does not bathe in maternal blood but in an exudate. Indeed, maternal spiral arteries are obstructed by intra-arterial cytotrophoblastic plugs, which block the entry of maternal blood into the intervillous space throughout the first trimester. The epithelium of endometrial glands is known to secrete material that is discharged into the lumen (uterine milk) [61, 62]. These secretions, a source of growth factors such as epidermal growth factor (EGF), VEGF, and leukaemia inhibitory factor, are delivered to the placental intervillous space. Fetal nutrition may thus be histiotrophic during this period [63, 64]. Angiogenin is a secreted protein present in amniotic fluid [65], follicular fluid [66], and plasma [67]. The punctuate angiogenin immunostaining of glandular epithelial cells from 7.5-week decidua points to the presence of secretory vesicles. At this time, the oxygen tension is <20 mm Hg [63]. Low but physiological oxygen tension may stimulate villous sprouting and capillary growth [15]. Angiogenin expression has been shown to be upregulated by hypoxic conditions in cultured cells, including granulosa cells [66], decidual cells [68], choriocarcinoma cells, and Simian virus 40 transformed placental cells [69]. Angiogenin might participate, along with other cytokines such as EGF, VEGF, and tumor necrosis factor-*α*, in regulating trophoblast proliferation and/or migration and might influence the remodelling of uteroplacental arteries.

Angiogenin was also expressed in decidual cells from first-trimester placenta, where it was strongly associated with the matrix which reminds that angiogenin is a heparin-binding protein [70] that binds to the extracellular matrix [54]. It has been shown that angiogenin is present in placental stromal and epithelial cells, with increased expression in the endometrium in the mid- and late secretory phases and early gestation [68]. In the decidua, we found that the uterine arteries and surrounding maternal macrophages also expressed angiogenin. The decidual environment is known to be immunosuppressive [71]. Angiogenin has been reported to function as an immunomodulator [72–74]. It is conceivable that angiogenin could thus participate in maternal immune tolerance towards the semiallogenic fetus.

Based on the other known biological activities of angiogenin and on its pattern of placental expression, our findings suggest that angiogenin, in concert with other regulators, may play a fundamental role in placental organogenesis.

## 5. Conclusions

This work shows that angiogenin is expressed throughout human placental development. In the early placenta, angiogenin is expressed by extravillous and villous cytotrophoblasts, as well as by functional syncytiotrophoblasts differentiated* in vitro*. A compatible pattern of angiogenin expression was observed* in situ*. Angiogenin expression in extravillous cytotrophoblasts* in vitro* and in the maternal decidua* in situ* (uterine glands, decidual cells, maternal artery and small capillaries, and macrophages) suggests that angiogenin may play a role in the decidual environment. The cellular pattern of angiogenin distribution in early first-trimester mesenchymal villi suggests a role in blood vessel formation. Fine analysis with cell-type-specific markers suggests a role of angiogenin in cross-talk between trophoblasts and endothelial cells. In view of its known biological activities, our findings suggest a fundamental role of angiogenin in early placental development.

## Figures and Tables

**Table 1:** Antibodies used in this study.

Antibody specificity	Species, isotype	Concentration used	Cell specificity in placenta	Source
Angiogenin	Rabbit IgG Mouse IgM Clone MANG-1	6 *μ*g/mL 6 *μ*g/mL	Aim of the study	[10] Bachem (San Carlos, CA)
Cytokeratin 7	Mouse IgG1 Clone OV-TL 12/30	2 *μ*g/mL	Trophoblastic cells	Dako (Glostrup, Denmark)
Pan cytokeratin (5, 6, 8, 17, and probably 19)	Mouse IgG1 Clone MNF116	0.9 *μ*g/mL	Epithelial glandular cells Trophoblastic cells	Dako
Vimentin	Mouse IgG1 Clone V9	5 *μ*g/mL	Endothelial cells, mesenchymal cells, and vascular smooth muscle cells Monocytes Macrophages	Immunotech (Marseille, France)
Alpha smooth muscle actin	Mouse IgG2a Clone 1A4	Ascites fluid 1/300	Vascular smooth muscle cells	Sigma ImmunoChemicals (St. Louis, MO)
Ki-67	Mouse IgG1 Clone MIB-1	4 *μ*g/mL	Proliferating cells	Immunotech
CD31, PECAM-1 (Platelet/endothelial cell adhesion molecule-1 )	Mouse IgG1 Clone JC/70A	4 *μ*g/mL	Endothelial cells	Dako
CD34	Mouse IgG1 Clone Qbend 10	4 *μ*g/mL	Endothelial cells Hematopoietic precursors	Immunotech
Tie-2 (Tyrosine kinase with immunoglobulin and epidermal growth factor homology domains-2)	Rabbit IgG	6 *μ*g/mL	Endothelial cells	Santa Cruz Biotechnology (Santa Cruz, CA)
VE-cadherin (Vascular endothelial cadherin), CD144, cadherin 5	Mouse IgG1 Clone TEA1/31	4 *μ*g/mL	Endothelial cells	Immunotech
VEGF-R2 (Flk-1) (Vascular endothelial growth factor receptor-2)	Mouse IgG1 Clone A3	4 *μ*g/mL	Endothelial cells	Santa Cruz Biotechnology
vWF (von Willebrand factor)	Mouse IgG1	4 *μ*g/mL	Endothelial cells	Roche Diagnostics (Meylan, France)
Epo-R (Erythropoietin receptor)	Rabbit IgG	6 *μ*g/mL	Endothelial cells Erythroid precursors	Santa Cruz Biotechnology
CD14	Mouse IgG2b Clone MY4	6 *μ*g/mL	Mature monocytes Macrophages	Immunotech
CD45 (Leukocyte common antigen)	Mouse IgG1 Clone HI30	6 *μ*g/mL	Leukocytes	BD Pharmingen (Le Pont-de-Claix, France)
Isotype control	Mouse IgG1		Nonspecific	Coulter Immunology (Hialeah, FL)
	Mouse IgG2a			Immunotech
	Mouse IgG2b			Coulter Immunology
	Mouse IgM			Coulter Immunology
	Rabbit IgG			Jackson ImmunoResearch Laboratories, Inc (West Grove, PA)
FITC-conjugated donkey anti-mouse IgG	Affinity Pure donkey IgG	3.5 *μ*g/mL		Jackson ImmunoResearch
Texas Red-conjugated goat anti-mouse IgG (subclasses 1 + 2a + 2b + 3)	Affinity Pure goat IgG, Fc fragment specific	3.5 *μ*g/mL		Jackson ImmunoResearch
FITC-conjugated goat anti-mouse IgM	Affinity Pure goat IgG, *μ* chain specific	3.75 *μ*g/mL		Jackson ImmunoResearch
FITC-conjugated goat anti-rabbit IgG	Affinity Pure goat IgG, Fc fragment specific	3.75 *μ*g/mL		Jackson ImmunoResearch
Rhodamine (TRITC-) labelled goat anti-rabbit IgG	Affinity Pure goat IgG, absorbed against human IgG	4.4 *μ*g/mL		Sigma BioSciences
Texas Red-conjugated donkey anti-rabbit IgG	Affinity Pure donkey IgG	3.5 *μ*g/mL		Jackson ImmunoResearch

**Table 2:** Primer sequences.

Gene	GenBank accession number	Primer sequences	Amplicon length	Reference
Human angiogenin	M11567	5′-CAT CAT GAG GAG ACG GGG-3′ sense, bp 1964–1981	264 bp	[21]
		5′-TCC AAG TGG ACA GGT AAG CC-3′ antisense, bp 2227 > 2208		

*β*-actin	M10277	5′-ACA ATG AGC TGC GTG TGG CT-3′ sense, bp 1496–1515	371 bp	After [22]
		5′-CTC CTT AAT GTC ACG CAC GAT TTC-3′ antisense, bp 2307 > 2284		

## Abbreviations

EGF: Epidermal growth factor
FGF: Fibroblast growth factor
hCG: Human chorionic gonadotropin
IgG: Immunoglobulin G
RNase: Ribonuclease
VEGF: Vascular endothelial growth factor.
